# The Influences of 1-Butyl-3-Methylimidazolium Tetrafluoroborate on Electrochemical, Thermal and Structural Studies as Ionic Liquid Gel Polymer Electrolyte

**DOI:** 10.3390/polym13081277

**Published:** 2021-04-14

**Authors:** Mariah Zuliana Dzulkipli, Jamilah Karim, Azizan Ahmad, Nurul Akmaliah Dzulkurnain, Mohd Sukor Su’ait, Masahiro Yoshizawa-Fujita, Lee Tian Khoon, Nur Hasyareeda Hassan

**Affiliations:** 1 Department of Chemical Sciences, Faculty of Sciences and Technology, Universiti Kebangsaan Malaysia, Bangi 43600, Malaysia; mariahzuliana@yahoo.com (M.Z.D.); jamilahkarim@nahrim.gov.my (J.K.); azizan@ukm.edu.my (A.A.); nurulakmaliah86@gmail.com (N.A.D.); 2 Department of Physics, University of Airlangga, Surabaya 60115, Indonesia; 3 Solar Energy Research Institute, Universiti Kebangsaan Malaysia, Bangi 43600, Malaysia; mohdsukor@ukm.edu.my; 4 Department of Materials and Life Sciences, Sophia University, 7-1 Kioi-cho, Chiyoda-ku, Tokyo 102-8554, Japan; masahi-f@sophia.ac.jp

**Keywords:** 1-butyl-3-methylimidazolium tetrafluoroborate, ionic liquid gel polymer electrolytes

## Abstract

After decades of development, ionic liquid gel polymer electrolytes (ILGPEs) are currently experiencing a renaissance as a promising electrolyte to be used in electrochemical devices. Their inherent tendency towards poor electrochemical properties have limited their applications and commercialization activities. Henceforth, gel polymer electrolyte (GPE) is being introduced to alleviate the abovementioned issues. In this work, the assessment of the ionic liquid 1-butyl-3-methylimidazolium tetrafluoroborate [BMIM][BF_4_] in poly (vinylidene fluoride-hexafluoropropylene) (PVDF-HFP) to form ILGPE was done. The relationship of [BMIM][BF_4_] towards the dielectric properties at different wt. % ratios and temperature was ascertained. The results indicated that [BMIM]BF_4_ is able to facilitate fast conduction. Moreover, it was found that [BMIM][BF_4_] could serve as an effective agent in reducing crystallinity and glass transition temperature of the polymer and thus enhanced the ionic conductivity of the samples. Notwithstanding, the ILGPE sample possessed a high thermal stability up to 300 °C and good electrochemical stability of 4.2 V which are beneficial for operation in electrochemical devices. All in all, the correlation between the ionic liquid chemistry and electrochemical performances could provide a valuable insight to rational selection and design for ILGPE electrolytes.

## 1. Introduction

The discovery of electrolytes was in the work of Alessandro Volta in the year 1800 when he invented the first electrochemical cell, which consisted of two dissimilar electrodes dipped in an electrolyte solution of saltwater brine, producing a steady electric current [1]. Ever since, various types of liquid electrolytes have been developed and were commercialized for use in electrochemical devices such as rechargeable batteries, solar cells, and fuel cell applications. Liquid electrolytes often achieve high ionic conductivity values due to high ion mobility in electrolytes, especially in less viscous electrolytes. Nonetheless, the use of liquid electrolyte still encounters several safety problems. It is highly volatile and flammable due to the use of organic solvents. In addition, liquid electrolyte represents a greater risk of leakage. These factors have resulted in severe combustion in some cases. For this reason, a tremendous research effort in search of a safer electrolyte has led to a solid-state electrolyte (SSE). Solid polymer electrolyte (SPE) is one of the most promising SSEs and comprises polymer and salt. The SPEs offer some distinct advantages over electrolytes dissolved in organic solvents. The use of SPE reduces the risk of leakage and thus somewhat mitigates the danger of toxic, corrosive, or flammable electrolytes and solvents especially in lithium-ion battery application [2]. For dye-sensitized solar cell (DSSC) application, SPE can avoid potential leakage, poor temperature stability and low potential. At low temperatures, the electrolyte can freeze and will cause a power disruption. This potentially leads to unwanted physical damages. At higher temperatures, the liquid electrolyte will expand and cause sealing damage to the panels. Notwithstanding, SPE is often used as membrane in proton exchange membrane fuel cell (PEMFC) application. PEMFC offers high energy efficiency (>80%), benign environment and reduced noise pollution comparing to diesel engines [3]. Other applications of polymer electrolyte based on ionic liquid can be found in solar-driven steam [4] and supercapacitor [5].

Over the past decades since the first pioneering work by Fenton and Wright on PEO-LiX salt in 1973 [6], most SPEs still possess poor ionic conductivity (10^−8^~10^−5^ S cm^−1^), which makes it unsuitable for practical applications [7]. Currently, gel polymer electrolyte (GPE), known as plasticized polymer electrolyte, has attracted great attention for its capability to mitigate the problems of liquid electrolyte while maintaining high ionic conductivity. The concept of GPE first started out by adding plasticizers with the purpose of reducing the crystallinity of polymers for better ionic conductivity [8,9]. Some of the plasticized used are low molecular weight aprotic solvent such as ethylene carbonate (EC), propylene carbonate (PC) or dibutyl phthalate (DBP) [10]. However, these conventional organic plasticizers are not suitable to be operated at high temperature and have insufficient mechanic strength [11,12]. Therefore, these have limited use in most electrochemical devices while some applications that need to be operated at high temperature require a high thermal stability electrolyte to ensure smooth operation and safety.

Thus, ionic liquid (IL) has become increasing popular choice to replace the use of low molecular weight plasticizers. The interest of IL as a “green” solvent lies in their extremely low vapor pressure, nonflammability, high designability, and high thermal stability (up to 300–500 °C). In term of electrochemical properties, they possess excellent ionic conductivity (typically 10^−2^–10^−3^ S cm^−1^) and wide electrochemical window (4–7 V) [13]. Owing to IL’s nature of being a self-dissociated ion and ability to dissociate salts, the ILs have shown to enhance the ionic conductivity due to the increased number of mobile charge carrier available for transport. Of all different classes of ILs, imidazolium cation-based IL are highly favorable for various industrial applications due to their remarkable solvating properties, low melting points, low viscosity as well as good air and water stabilities [14,15].

Generally, poly(ethylene oxide) (PEO), polyacrylonitrile (PAN), poly(methyl methacrylate) (PMMA), poly(trimethylene carbonate), poly(vinylidene fluoride) (PVDF), and poly(vinylidene fluoride-hexafluoropropylene) (PVDF-HFP) are among the common solid media used as a polymer host in the electrolytes field [7,10,16,17,18]. However, PVDF-HFP is one of the most well-sought-after polymer hosts for ionic liquid gel polymer electrolytes (ILGPEs) due to its excellent film formation ability, hydrophobicity, high mechanical and thermal stability. Furthermore, PVDF-HFP is a semicrystalline polymer and possesses both crystalline -VDF and amorphous -HFP units. The crystalline phase of PVDF provides the mechanical support whereas the amorphous phase is capable of trapping large amount of electrolyte [19]. Apart from that, its high dielectric constant of *ε* = 8.4 and high electrochemical stability due to the presence of strong electron withdrawing group of (–C–F) also gives positive contribution to its overall electrochemical performance [20,21,22]. Nevertheless, the high crystallinity in PVDF-HFP impedes the mobility of the ions; thus, it is only capable of achieving an average ionic conductivity value. Often, the PVDF-HFP films are stiff and have poor interfacial contact with electrodes. In order to overcome the drawbacks, a good combination with IL is required, depending on the choice of applications. For example, high-temperature PEMFC application requires a high thermal stability of protic IL whereas lithium-ion battery often use aprotic IL with low viscosity to enhance its room temperature ionic conductivity.

Motivated by the ILs advantages, we seek to improve the PVDF-HFP properties by incorporating 1-butyl-3-methylimidazolium tetrafluoroborate [BMIM][BF_4_] which will serve as both the ion source and plasticizer in this present work. The [BMIM][BF_4_] has room temperature conductivity of ~3 mS cm^−1^ and electrochemical stability of ~4.0 V. Furthermore, it does not have a melting point but low glass transition temperature at ca. −85 °C and viscosity value of 219 cP (25 °C), whereas BMIMPF_6_ has higher T_g_ (−76 °C) and viscosity (450 cP at 25 °C) [23,24]. These are good requirements when it comes to LIB application which required low viscosity.

Herein, this paper demonstrates the improvement of ILGPE based PVDF-HFP using [BMIM]BF_4_ ionic liquid. Studies on effects of electrical, electrochemical, thermal, and structural properties of the polymer electrolytes when adding high amounts of ionic liquid in the system will be shown. In addition, we present a facile ILGPE preparation of stable ILGPE films with a high loading amount of ionic liquid (>50%) through solution casting. It exhibited good electrochemical and thermal properties, which we believe could act as a potential electrolyte to be applied in electrochemical devices.

## 2. Experimental

### 2.1. Materials

Poly(vinylidene fluoride-hexafluoropropylene) (PVDF-HFP) (purity > 99.9%) in pellet form and 1-butyl-3-methylimidazolium tetrafluoroborate [BMIM][BF_4_] (purity > 98.0%) (*M*_w_ = 226.02 g/mol) were purchased from Sigma-Aldrich (St. Louis, MO, USA). Acetone solvent (purity > 99.8%) (*M*_w_ = 58.08 g/mol) (Grade: ACS, ISO, Reag. Ph Eur) was obtained from Merck (Kenilworth, NJ, USA). Unless otherwise specified, reagents and chemicals were used as received without further purification.

### 2.2. Preparation of Ionic Liquid Gel Polymer Electrolyte

ILGPE was prepared by dissolving an appropriate amount of PVDF-HFP in 10 mL of acetone solvent and stirred for 24 h at room temperature. Afterward, [BMIM][BF_4_] as a plasticizing agent was added and the mixture were further stirred for 24 h to achieve a homogenous solution. The electrolyte solution was then cast onto a Teflon dish and the solvent was let to evaporate slowly for 48 h. The dried gel was peeled off and a free-standing and flexible film was attained. The obtained ILGPE films were further dried in vacuum oven to ensure that it is free from solvent and moisture, then stored in a desiccator until further analysis. The above samples were prepared at different ratios to PVDF-HFP:[BMIM][BF_4_] using the same procedures.

### 2.3. Characterizations

Dielectric studies were conducted by extracting the impedance data measured using a potentiostat model VersaSTAT 4 by Princeton Applied Research (Oak Ridge, Tennessee, USA) at room and elevated temperature. Prior to analysis, ILGPE films were punched into small disc shape with an area of 2.01 cm^2^. The measurement was performed in a stainless steel (SS) holder with the configuration of SS/ILGPE film/SS. The whole assembly was kept in a temperature-controlled oven and the measurement was taken in the temperature range of 303 K to 373 K. Dielectric properties of the ILGPE films; dielectric constant ($\varepsilon^{\prime }$) and dielectric loss ($\varepsilon^{\prime \prime }$) were calculated using the following equations.

(1) $$\varepsilon^{\prime }=Z_{i}/\omega C_{0}\left(Z_{r}{}^{2}+Z_{i}{}^{2}\right)$$

(2) $$\varepsilon^{\prime \prime }=Z_{r}/\omega C_{0}\left(Z_{r}{}^{2}+Z_{i}{}^{2}\right)$$

where $Z_{i}$ and $Z_{r}$ are the imaginary and real impedance, respectively. Angular frequency, ω=2πf, where f is the frequency, and $C_{0}=\varepsilon_{o}A/l\;$in which $\varepsilon_{o}$ = permittivity of free space, A = effective contact area of electrolyte and the electrode, and l = thickness of the sample. Further analysis from the impedance data such as dielectric modulus and loss tangent (tanδ) were carried out. Dielectric modulus, which contains two parts, real ($M^{\prime }$) and imaginary ($M^{\prime \prime }$), were calculated using the equations that follow:

(3) M′=ε′(ε′2+ε″2)

(4) M″=ε″(ε′2+ε″2)

Meanwhile, the ratio between $\varepsilon^{\prime \prime }$ and $\varepsilon^{\prime }$ quantified as loss tangent (tanδ) was expressed below:

(5) tanδ=ε″ε′

Electrochemical stability window of the highest conducting film was determined using Wonatech ZIVE MP2 Multichannel electrochemical workstation (Seoul, Korea). ILGPE film was placed in between two stainless steel blocking electrodes and the analysis was carried out from 0 to 5 V with scan rate of 1 mVs^−1^. Thermal characteristics of ILGPE films were investigated using DSC 8822^e^ Mettler Toledo (Greifensee, Switzerland). Two cycles of heating were performed on each film. The first cycle began from −50 to 100 °C whilst the second cycle was heated up to 200 °C with a heating rate of 10 °C min^−1^. Thermogravimetric analyzer, which is Mettler-Toledo TGA/SDTA 851 was used to determine the thermal stabilities of ILGPE films in the temperature range of 25 °C to 800 °C under inert atmosphere at a heating rate of 10 °C min^−1^. The films were carefully weighed in the range of about 7.0 mg. The crystallinity of the ILGPE films were investigated using XRD D5000 Siemens (Munich, Bavaria, Germany). Diffraction angle, 2*θ,* was taken from 3° to 60° at a rate of 0.05° s^−1^.

## 3. Results and Discussion

### 3.1. Physical Observation

**Figure 1:**
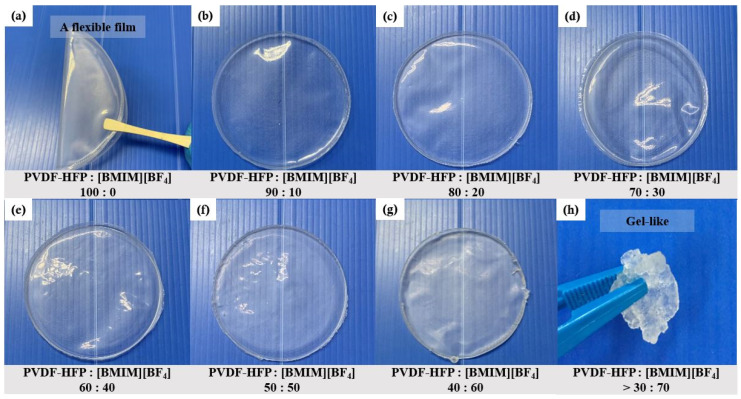
Physical observation of PVDF-HFP:[BMIM][BF_4_] (**a**) 100:0, (**b**) 90:10, (**c**) 80:20, (**d**) 70:30, (**e**) 60:40, (**f**) 50:50, (**g**) 40:60 and (**h**) >30:70.

Figure 1 shows the physical observation of ILGPE films at various percentage ratios of PVDF-HFP to [BMIM][BF_4_]. Generally, all the films display good homogeneity despite mixing two different types of entities. This is reflected through good transparency, softness, and flexibility as shown in Figure 1. However, at higher rations of [BMIM][BF_4_], the samples were mechanically unstable and became gel-like. In accordance with the intended purpose, samples above the ratio of 60:40 will not be considered for further examination and study.

### 3.2. Dielectric-Temperature-Dependent Studies

Ionic conductivity is one of the key indicators for high-performance electrolyte in any electrochemical devices. For example, PEMFCs and LIBs applications demand a minimum ionic conductivity of 10^−3^ S cm^−1^ to be functioned. The influences of [BMIM][BF_4_] on conductivity and conduction behavior of the ILGPEs have been discussed in our previous work [25]. At optimum ratio of 40:60 (PVDF-HFP to [BMIM][BF_4_]) sample, the highest conductivity of 2.84 × 10^−3^ S cm^−1^ with transference number (TN) value of 0.998 are obtained. The near unity value of 1 in TN indicates ion conduction is primary predominately by ions and not electron conduction [26,27]. Thereby, the studies on dielectric properties will provide an important insight on the polarization effect at the electrode–electrolyte interfaces, ion conduction behavior and relaxation mechanism of the electrolyte system. In the field of polymer electrolytes, dielectric constant (ε’) is a measure of the polymer electrolyte’s capacity to store charge carriers and it represents the amount of dipole alignment in a given volume. On the other hand, dielectric loss (ε’’) is a measure of energy loss due to the motion of free charge carriers and dipoles alignment when the polarity of electric field reverses rapidly [28]. All the dielectric and modulus data are calculated using the data collected from impedance spectroscopy (IS) which can be found in our previous work [26].

**Figure 2:**
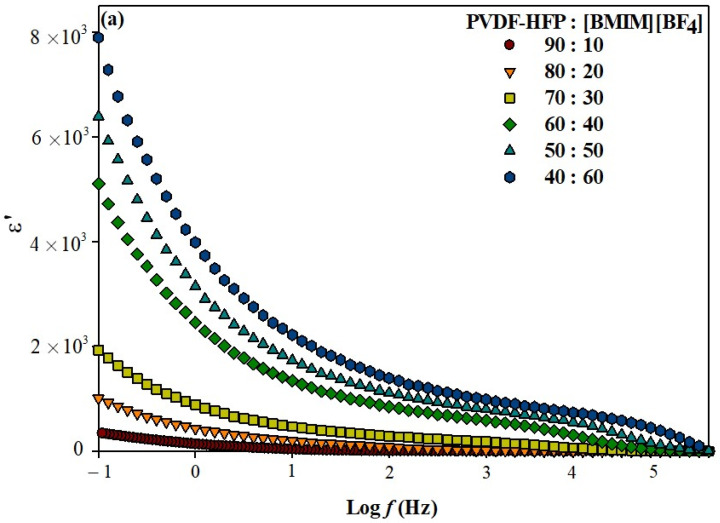

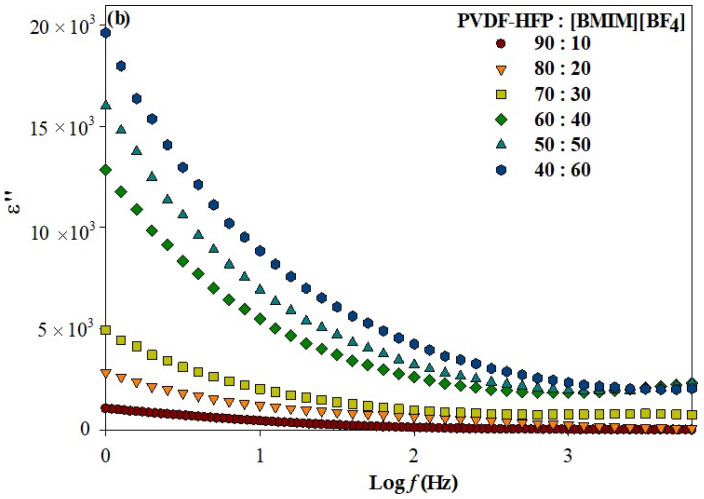
Frequency dependence of (**a**) dielectric constant and (**b**) dielectric loss for different percentage ratios of PVDF-HFP to [BMIM][BF_4_].

Figure 2 shows the graph of frequency dependence of $\varepsilon^{\prime }$ and $\varepsilon^{\prime \prime }$ at various PVDF-HFP to [BMIM][BF_4_] contents. It is observed from both Figure 2a and b that $\varepsilon^{\prime }$ and $\varepsilon^{\prime \prime }$ display similar pattern in which there is an increasing peak at the lower frequency region and a gradual decrease at the higher frequency region for all ILGPEs. The high value of $\varepsilon^{\prime }$ observed at low frequency region is due to the accumulations of charge at electrode-electrolyte interface resulted from slow periodic reversal effect of the applied field [29]. Whereas, at the higher frequency region, the periodic reversal of the applied field occurred so fast that the charge carriers and dipoles in the polymer chain are incapable to cope up with the speed and orient themselves according to the direction of the applied field. Hence, the value of $\varepsilon^{\prime }$ at high frequency region is small as there is no excess of ion diffusion [30,31]. Apart from that, the $\varepsilon^{\prime }$ value increased with increasing percentage ratio of [BMIM][BF_4_]. This insinuates that the charge carrier density increases with higher IL loading in the ILGPE system. A similar trend has also been reported for other polymer electrolyte systems [32,33].

**Figure 3:**
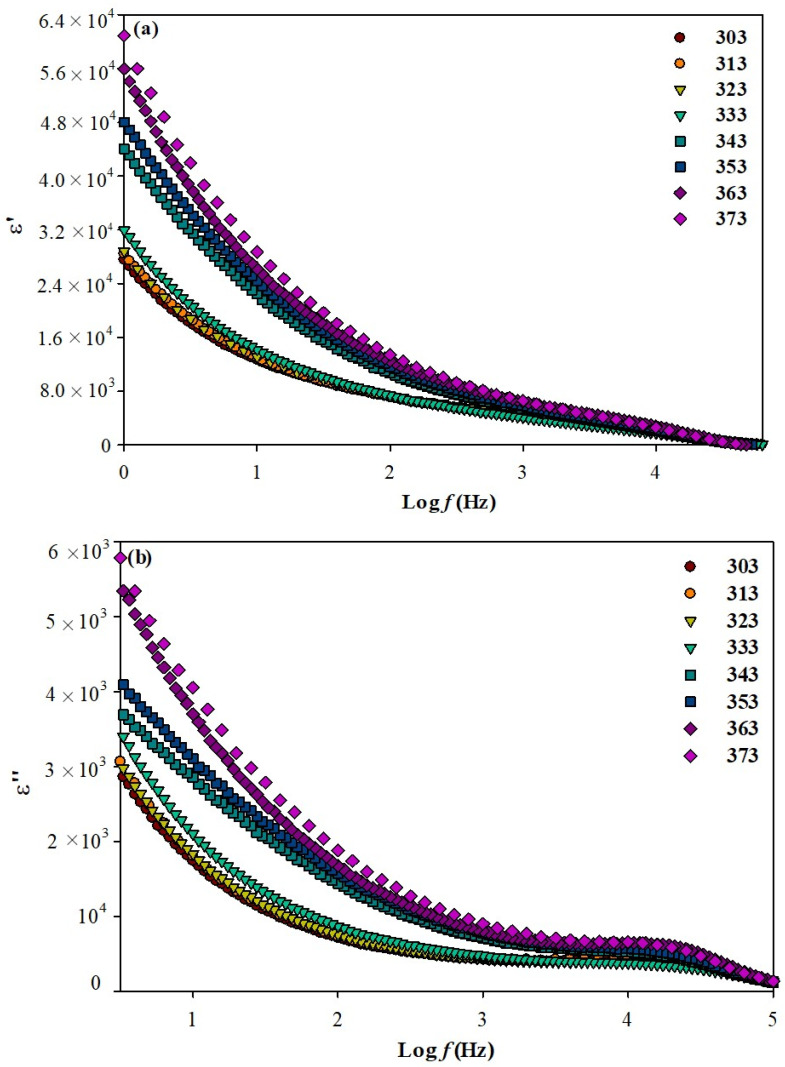
Frequency dependence of (**a**) dielectric constant and (**b**) dielectric loss at various temperatures for the highest conducting ILGPE film with 60% percentage ratio of [BMIM][BF_4_] to PVDF-HFP.

Figure 3a,b represents the plots of $\varepsilon^{\prime }$ and $\varepsilon^{\prime \prime }$ for the highest conducting film with a 60:40 ratio at various temperatures (303 K to 373 K). Based on both figures, it can be seen that the values of $\varepsilon^{\prime }$ and $\varepsilon^{\prime \prime }$ gradually increase with temperature. Presumably, the increase of temperature may increase the free volume and mobility of polymer segments, which facilitates the dipole orientation due to the greater movement of the dipole molecular chain of ILGPE [28,33].

**Figure 4:**
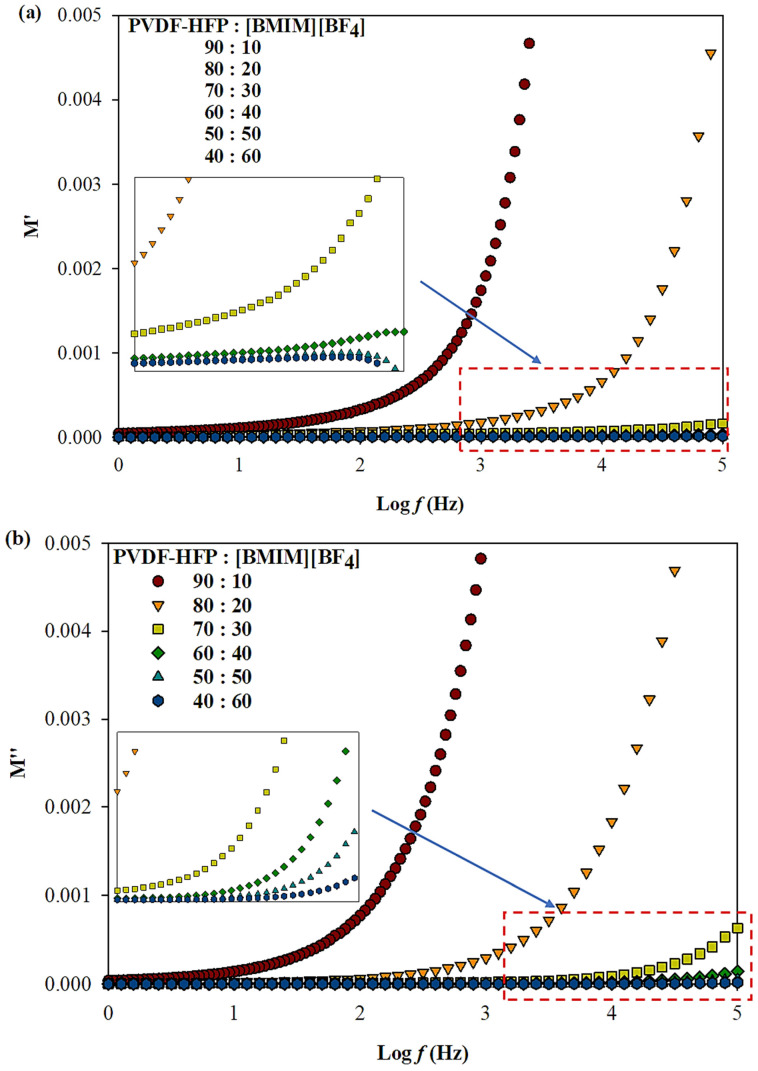
Frequency dependence of (**a**) real part and (**b**) imaginary part of modulus spectra for different percentage ratio of PVDF-HFP to [BMIM][BF_4_] at room temperature.

Further interpretation of the impedance data was carried out through dielectric modulus which study the behavior of electrode–electrolyte polarization effect at different frequencies. Figure 4a,b display the dielectric modulus of real ($M^{\prime }$) and imaginary modulus part ($M^{\prime \prime }$) as a function of frequency for the samples with different percentage ratios of PVDF-HFP to [BMIM][BF_4_]. The $M^{\prime }$ and $M^{\prime \prime }$ are calculated based on equation (3) and (4), respectively. As the frequency increases, $M^{\prime }$ and $M^{\prime \prime }$ also increased due to the distribution of the relaxation process and attain maximum value in the higher frequency range. From both figures, $M^{\prime }$ and $M^{\prime \prime }$ were observed to have peak dispersion in the high frequency region and decreases towards the low frequency region. The peaks at high frequencies highlighted on the bulk effect revealed that the ILGPE films are predominantly ionic conductors [34]. As the loading of IL increases, the spectra are shifted to the right beyond the frequency range used in this experiment indicating that the ILGPE film are purely an ionic conducting system. On the contrary, $M^{\prime }$ and $M^{\prime \prime }$ values approaching zero in the low frequency region are associated to the electrode–electrolyte polarization effect. Generally, the polarization effect at electrode–electrolyte interfaces is often suppressed at lower frequency, giving small values (tending zero). This indicates the removal of electrode polarization [35]. The plots that display a long tail close to the x-axis are due to large capacitance value that correlates with the large polarization effect at the electrode–electrolyte interface [27]. Therefore, the loading of 60% [BMIM][BF_4_] incorporated into the system proves that the films are purely due to ionic conductors.

**Figure 5:**
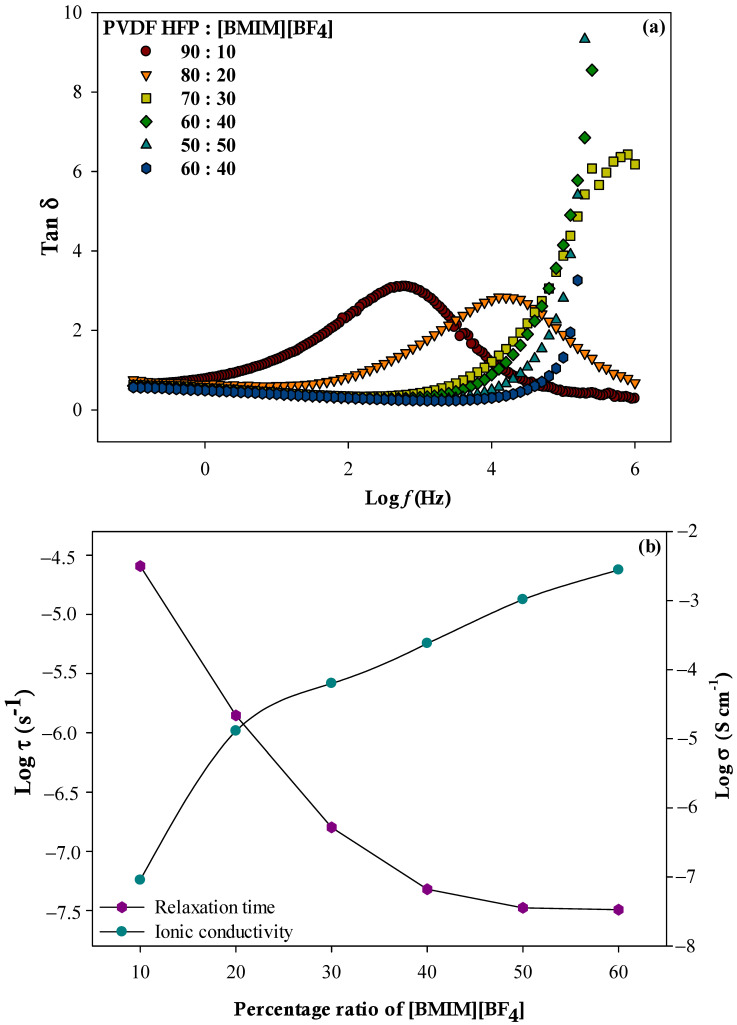
(**a**) Frequency dependence of tan *δ* and (**b**) relation between relaxation time and ionic conductivity for different percentage ratios of PVDF-HFP to [BMIM][BF_4_].

Analysis of loss tangent (tanδ) was carried out to observe the effect of IL loading towards the relaxation behavior. Variation of tanδ as a function with frequency for different percentage ratios of PVDF-HFP to [BMIM][BF_4_] is presented in Figure 5a. Tanδ or also known as dissipation factor, is a measurement of the ratio of energy loss to energy stored determined by the expression in equation (6). The occurrence of peak maxima of tanδ seen in the figure imply the presence of relaxation dipoles in the system [36]. It can be seen that the peak has shifted towards the high frequency region as the loading of [BMIM][BF_4_] is increased. At the same time, it also indicates the reduction of relaxation time. The relation between the relaxation time (τ) and frequency ($f_{max}$) corresponding to the maximum frequency peak expressed as below:

(6) $$\tau_{\left(tan\delta \right)}=1/\omega =1/2\pi f_{max}$$

The relaxation time is associated with the change diffusion process in the sample and is correlated with the plateau of the real part of the conductivity [31]. The reduction in relaxation time implies that the polymer chain can easily be oriented through the increment of free volume due to the incorporation of more amounts of ions. Presented in Figure 5b is the graph of relaxation time and ionic conductivity at various loading of PVDF-HFP to [BMIM][BF_4_]. It can be seen that the relation between relaxation time and ionic conductivity at various IL loadings is the opposite. As the ionic conductivity increases, the relaxation time decreases. This shows that increasing the loading of [BMIM][BF_4_] into the ILGPE system resulted in increased ionic conductivity which then helped in reducing the relaxation time during ionic movement in the sample [37]. Along with that, increase in height of tanδ for each ILGPE film also suggested the increment in charge carrier density owed to the increase of [BMIM][BF_4_] [38].

### 3.3. Electrochemical Stability Study—Linear Sweep Voltammetry

**Figure 6:**
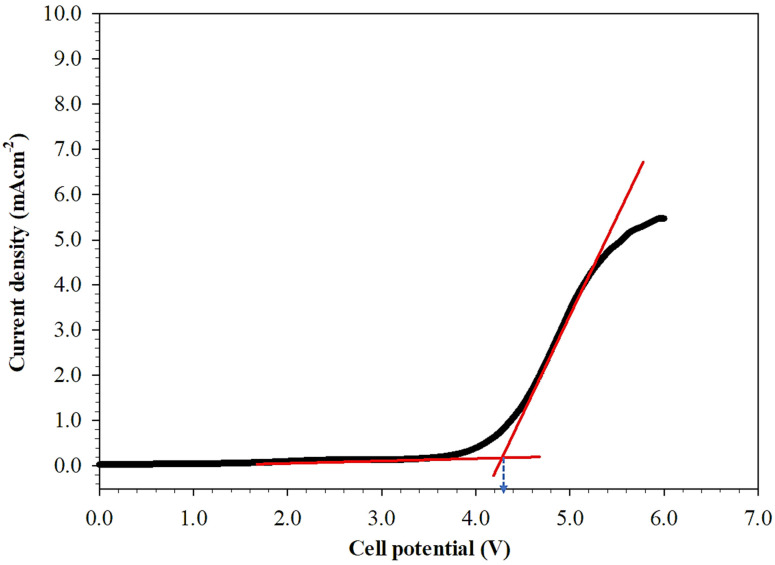
Linear sweep voltammetry of the highest conducting film containing 60% of [BMIM][BF_4_] to PVDF-HFP.

Electrochemical stability window (ESW) of the electrolyte plays a vital role especially when being applied in electrochemical device applications. ESW of the ILGPE film was measured by examining the breakdown voltage through LSV analysis [39]. The measurement was conducted using inert symmetrical electrode SS/ILGPE/SS cell configuration. Figure 6 shows the voltammogram of the optimum ILGPE sample at a scan rate of 1 mVs^−1^. As presented in the Figure 6, it is found that the breakdown voltage of the ILGPE occurs at 4.2 V. Similar result was obtained in the previous work by TianKhoon et. al, whereby the sudden change of anodic current occurred at around 3.8 V which indicates the start of the oxidation of [BF_4_]^−^ anions [40]. Studies conducted by Kazemiabnavi et al. revealed that anions that are highly fluorinated such as BF_4_^−^ tend to be electrochemically more stable against reduction and oxidation as compared to other anions [41]. As a result, fluorinated anions have wider ESW when coupled with an ionic liquid cation. In addition, the high stability value of 4.2 V is in good agreement with the previously reported value by Kazemiabnavi et al. and it also meets the requirement as a potential electrolyte for use in LIBs and PEMFC applications [41].

### 3.4. Thermal Analysis (DSC and TGA)

#### 3.4.1. Differential Scanning Calorimetry

**Figure 7:**
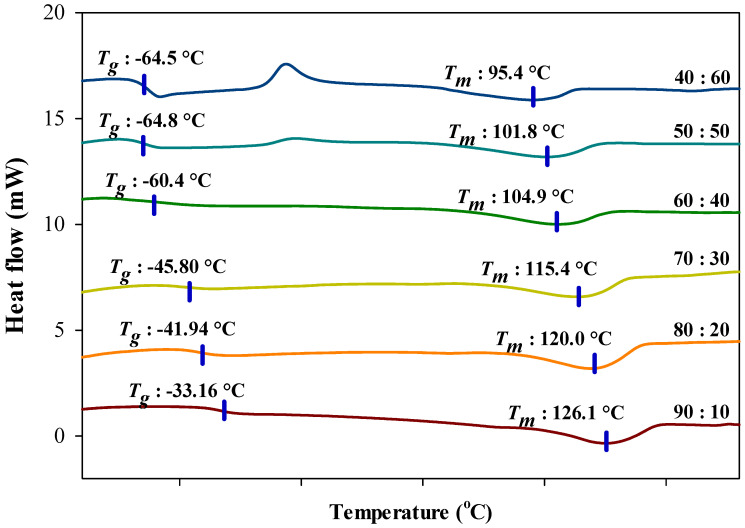
Differential scanning calorimetry (DSC) curves in the temperature range −100 °C to 180 °C for different percentage ratios of PVDF-HFP to [BMIM][BF_4_].

DSC analysis presents the essential information on the thermal characteristics of the electrolyte such as glass transition temperature ($T_{g}$), melting temperature ($T_{m}$) as well as the crystallization temperature ($T_{c}$). Figure 7 depicts the heat flow curves in the temperature range of −100 °C to 180 °C for different percentage ratios of ILGPEs and Table 1 provides the thermal properties of reference for PVDF-HFP and [BMIM][BF_4_] [22,42,43,44,45]. In ensuring the ILGPE films are completely free from any remnant trace of solvent and trapped moisture, all DSC traces were taken during the second heating. Based on the graph shown in Figure 7, it can be seen that upon higher incorporation of [BMIM][BF_4_], $T_{g}$ and $T_{m}$ values for all films shifted to lower temperature region, from −33.16 °C to −73.78 °C and 126.13 °C to 100.67 °C, respectively [46]. Along with that, there is an appearance of exothermic crystallization peak at higher percentage ratio, notably at 50:50 and 40:60 PVDF-HFP to [BMIM][BF_4_] which has also shifted to lower temperature; −3.39 °C and −6.54 °C. The shifting indicates that there is an interaction occurred between the polymer matrix, PVDF-HFP and ionic liquid [BMIM][BF_4_]. From the results, it shows that with further incorporation of [BMIM][BF_4_], $T_{g}$ of ILGPE films have decreased and inclined towards the $T_{g}$ of pure [BMIM][BF_4_]; −84 °C. This expresses the dominant influence of [BMIM][BF_4_] in the polymer matrix and that the plasticizing effect of the IL have weaken the intermolecular interactions between the polymer chains [47]. The weakening of the forces increases the flexibility of the polymer backbone and therefore, give rise to the ionic conductivity of the ILGPE [48,49]. Meanwhile, the shifting of $T_{m}$ to a lower temperature region suggests that there are more amorphous phases present in the ILGPE system. Pandey et al. [50] reported that a broader $T_{m}$ peak is attributed to the presence of liquid components that cause an increase in the amorphous proportion in the overall material.

#### 3.4.2. Thermogravimetric Analysis

**Figure 8:**
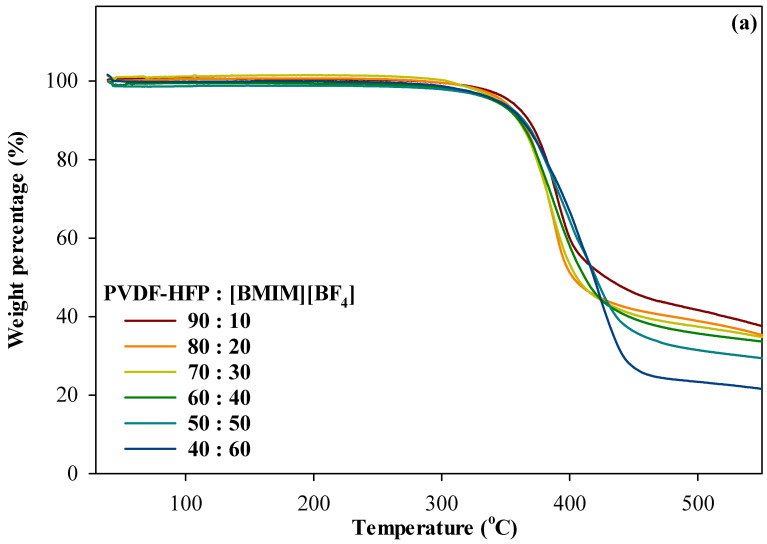

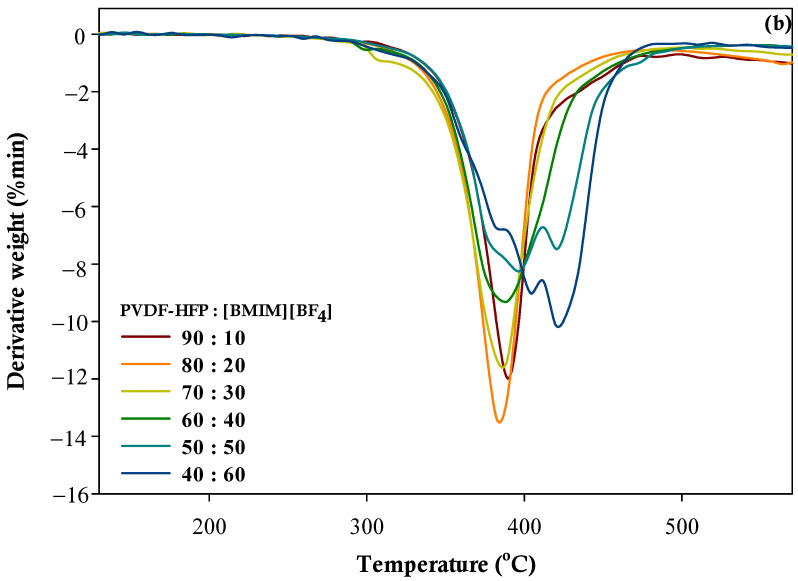
Thermogram of (**a**) TGA and (**b**) DTG of different percentage ratio of PVDF-HFP to [BMIM][BF_4_].

Thermal stability of an electrolyte is extremely vital to guarantee acceptable performance in any electrochemical devices and seemed to be a critical issue over the past decades. It is of great importance to develop an electrolyte with high thermal stability which can enhance the safety property in energy storage and conversion devices. For example, low-temperature PEMFC and high-temperature PEMFC operate in a range of 60–80 °C and 110–180 °C, respectively. Meanwhile, LIBs have typical temperature working range of −20–60 °C. Figure 8 depicts thermogram of TGA and differential gravimetric analysis (DTG) of the ILGPE films with different percentage ratio of PVDF-HFP to [BMIM][BF_4_]. According to the previous report, thermal degradation temperature ($T_{d}$) for pure PVDF-HFP is 470 °C whilst $T_{d}$ for [BMIM][BF_4_] is in the range of 360 °C to 420 °C [51,52]. TGA curves for all films showed high thermal stabilities up to 300 °C in which it is a major desirable property for the application of any electrochemical devices. No weight loss observed below 200 °C for all ILGPE films revealed that the sample is free from solvent and moisture. This is in good agreement with the hydrophobic nature of the fluoropolymer and moisture stable of imidazolium-based IL [10]. The first degradation step, which is in the range of 350 °C to 420 °C is believed due to the degradation of IL, since the $T_{d}$ of [BMIM][BF_4_] is lower compared to PVDF-HFP. Meanwhile, the following step from 420 °C to 465 °C may be due to the degradation of its polymer host [51]. The maximum weight loss percentage of the ILGPE is also in accordance with the loading of [BMIM][BF_4_] in the system.

The DTG peak in Figure 8b shows a sharp peak of degradation temperature ($T_{d}$) for each film. Incorporation of 10% to 30% [BMIM][BF_4_] showed only one peak at $T_{d1}$ ranging from 370 °C to 390 °C which was due to the degradation of [BMIM][BF_4_] as mentioned before. At higher incorporation of [BMIM][BF_4_] of 50% and 60%, there is another peak with $T_{d2}$ at 419 °C. This peak corresponds to the degradation of PVDF-HFP [51]. From the results, the decrease in thermal stability proves that polymer matrix and IL incorporated well with each other and was still in the right temperature working range that is suitable for most applications in electrochemical devices, in particular at high temperature.

### 3.5. Structural Analysis (X-Ray Diffractometry)

**Figure 9:**
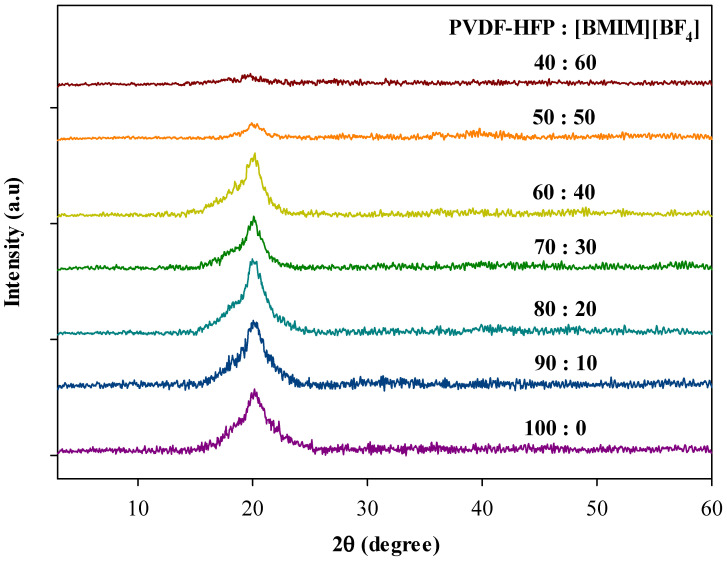
Diffractogram of pure PVDF-HFP and different percentage ratio of PVDF-HFP to [BMIM][BF_4_].

XRD was performed to determine crystalline state of each ILGPE film. Figure 9 represents the XRD patterns of pure PVDF-HFP and ILGPE films with different percentage ratios of PVDG-HFP to [BMIM][BF_4_]. The semicrystalline structure of pure PVDF-HFP film is confirmed by the coexistence of crystalline peaks (18° to 20°) in the amorphous region (15° to 25°) [53]. Upon addition of [BMIM][BF_4_], the prominent crystalline peak at 20° diminished and ceased to be visible, indicating that IL is an effective plasticizer for the PVDF-HFP polymer [50]. Besides, the degree of crystallinity (DC) for pure PVDF-HFP is found to be 59%. With as much as 10 wt.% of [BMIM][BF_4_] incorporated into the system, the DC decreased to 46.4% and subsequently reduced to 35.6% at 60 wt.% of IL loading. This implies that [BMIM][BF_4_] is not just as a charge carrier, but it also plays role as a plasticizer and thus, lowers the crystallinity as well as results in increased ionic conductivity of the ILGPE system [53].

## 4. Conclusions

Overall, PVDF-HFP incorporated with ionic liquid [BMIM][BF_4_] gel polymer electrolytes have substantially improved. These improvements have been reflected in the dielectric constant, dielectric loss, and tangent loss values of the ILGPE films that were found to increase upon addition of [BMIM]BF_4_. The values are higher when the percentage ratios of [BMIM][BF_4_] to PVDF-HFP increase, due to the increase in the number of charge carriers supplied by [BMIM][BF_4_]. The appearance of peaks in the modulus formalism spectra at high frequencies implies that the ILGPE films are predominantly ionic conductors. The DSC and XRD results showed that the crystallinity of ILGPE film significantly decreased with [BMIM][BF_4_]. The reduction of crystallinity helps by increasing of ionic conductivity. Meanwhile, all ILGPE films have high thermal stability up to 300 °C and lower weight loss which signify the improvement in thermal stability. Further investigation of the electrochemical stability carried out through LSV analysis revealed high ESW value of 4.2 V. In general, our findings showed that [BMIM][BF_4_] could serve as a reinforced agent to replace the volatile and flammable organic solvent for better GPE performance. Nevertheless, future works need to further study the compatibility of ILGPE-based PVDF-HFP/[BMIM]BF_4_ with electrodes and the solubility of salts such as LiTFSI for application in LIB.

## Figures and Tables

**Table 1:** Glass transition and melting temperature value for pure PVDF-HFP and [BMIM][BF_4_].

Pure Sample	$T_{g}$	$T_{m}$	Reference
PVDF-HFP	−35 °C	143 °C	[22,42]
[BMIM][BF_4_]	−84 °C	−75 °C	[43,44,45]

## Data Availability

The data presented in this study are available on request from the corresponding author. The data are not publicly available due to privacy restrictions.
